# RELIGIOUS ATTENDANCE, SOCIAL SUPPORT, AND SELF-RATED HEALTH IN FORMER NATIONAL FOOTBALL LEAGUE ATHLETES

**DOI:** 10.1093/geroni/igz038.2306

**Published:** 2019-11-08

**Authors:** Robert W Turner II, Robert Turner, Amanda Sonnega, Tim Cupery, Evelyn Bush, Teri Rosales, James S Jackson

**Affiliations:** 1 The George Washington University, Washington, District of Columbia, United States; 2 Center on BioBehavioral Health Disparities Research, Duke University, Durham, North Carolina, United States; 3 Institute for Social Research, Ann Arbor, Michigan, United States; 4 Fresno State, Fresno, California, United States; 5 Fordham, Bronx, New York, United States; 6 University of Michigan, Ann Arbor, Michigan, United States

## Abstract

Concern exists about the health and well-being of football players, yet little research exists on the psychosocial risk and protective factors of NFL athletes’ well-being. This study assesses the role of religious attendance, social support, and self-rated health in former NFL athletes. Data comes from a stratified, random sample of 1,063 former NFL players. A set of nested linear regression models evaluated the relationship between self-rated health status and two indices of social support (family and friends) and attendance at religious services. Frequent attendance at religious services (β=0.19, p<.01), support from family (β=0.06, p<.05), and support from friends (β=0.06, p<.01) are positively and significantly related to better self-rated health. The ability to get out of the house did not affect these associations. However, the pain symptoms index fully accounted for any positive effect of family support and religious attendance in self-rated health.

